# Evaluation of the diagnostic utility of galectin-1 and TROP-2 in thyroid tumors: A retrospective analysis

**DOI:** 10.5339/qmj.2025.113

**Published:** 2025-12-11

**Authors:** Methaq Mueen Al-Kaabi, Mohanad Mahdi Al-Hindawi, Ikram A. Hasan

**Affiliations:** 1 Department of Pathology and Forensic Medicine, College of Medicine, Mustansiriyah University, Baghdad, Iraq; 2 Department of Pathology and Forensic Medicine, College of Medicine, University of Kerbala, Karbala, Iraq *Email: mohanad.m@uokerbala.edu.iq

**Keywords:** trophoblast cell surface antigen 2 (TROP-2), galectin 1, thyroid neoplasms

## Abstract

**Background:**

Thyroid cancer is the most common endocrine cancer worldwide. Differentiation between benign and malignant thyroid pathology is crucial for optimum management. Immunohistochemical markers (IHC) such as galectin-1 and the trophoblast cell surface antigen 2 (TROP-2) are valuable tools in this differentiation. The study aimed to evaluate the role of immunohistochemical expression of galectin-1 and TROP-2 in differentiating benign from malignant thyroid lesions.

**Methodology:** This retrospective observational study analyzed 136 thyroid specimens, including 50 cases of follicular nodular disease (FND), 23 follicular adenomas (FA), 37 papillary carcinomas (PTCs), and 26 follicular carcinomas (FTCs) collected from Al-Yarmouk Teaching Hospital from October 2022 to October 2024. All cases underwent IHC staining for galectin 1 and TROP-2.

**Results:**

Galectin-1 IHC expression was significantly associated with tumor size of papillary carcinoma cases (*P* = 0.002). TROP-2 IHC expression was significantly associated with female gender (*P* = 0.0006) and tumor size in follicular carcinoma cases (*P* = 0.031). A highly significant difference in IHC expression between benign and malignant cases was observed for both markers (*P* = 0.001). TROP-2 expression was higher in malignant cases at 82% (papillary and follicular carcinoma) compared to galectin-1 (62%; *P* value, 0.0097).

**Conclusion:**

Both galectin-1 and TROP-2 showed higher expression in malignant thyroid cases compared to benign lesions. TROP-2 showed higher sensitivity in detecting malignant lesions, whereas galectin-1 exhibits greater specificity. Combining both markers enhances the differentiation between benign and malignant lesions, thus providing valuable diagnostic insight.

## 1. INTRODUCTION

Thyroid cancers are the most common endocrine cancer worldwide. Its incidence is increasing due to the improvement of diagnostic procedures.^1^ According to the Global Cancer Observatory (GLOBOCAN) 2022, more than 821 thousand cases of thyroid carcinoma were diagnosed globally, with an age-standardized incidence rate of 9.10 per 100,000 population. Women represented about 75% of these newly diagnosed cases, with most individuals being in the 40 to 50-year age group.^2^

According to the World Health Organization (WHO) classification, thyroid malignancy is classified into papillary thyroid carcinoma, which represents 80% of thyroid cancer, follicular carcinoma, medullary carcinoma, poorly differentiated and anaplastic carcinoma.^3^

The gold standard method for diagnosing various thyroid lesions is still histopathological examination using routine hematoxylin and eosin (H&E) stain; however, some cases are challenging in diagnosis, especially those with ambiguous morphological features. Therefore, the application of immunohistochemistry (IHC) may serve as an adjunct method for diagnosis.^4^

Many immunohistochemical markers were employed for this purpose, individually or in combination. Some commonly used markers include those for determining cell of origin, like thyroglobulin (TG), thyroid transcription factor-1 (TTF-1), and calcitonin. Others are used for differentiating benign from malignant neoplasms with cytokeratin 19 (CK19), Hector Battifora mesothelial cell-1 (HBME-1), galectin-3, and CD56 being mostly tested; however, these markers showed variable sensitivity and specificity, necessitating the need for newer, more helpful markers.^5^

Galectins are a family of animal lectins that share a common sequence of amino acids and the carbohydrate recognition domain (CRD) with high affinity for β-galactosidase and are important in carbohydrate recognition. They are considered proteins with carbohydrate-binding abilities that cross-bind bound with glycoconjugates on different receptors, leading to different signaling pathways. Galectins are involved in the regulation of many cellular functions, like cell proliferation, adhesion, migration, pathogen recognition, antigen processing, and phagocytosis. Many studies revealed that galectins may affect different steps of cancer development through the recruitment of immune cells like neutrophils, lymphocytes, and monocytes to the inflammatory sites and modulating their functions. Galectins play a pivotal role in tumor progression and metastases by facilitating interaction between the tumor and the tumor microenvironment. Dysregulation of galectin gene expression by cancer may participate in the distinctive features of cancers, like apoptosis resistance, malignant transformation of cells, angiogenesis, and tumor metastasis.^6^

Galectins are a family of about 15 proteins. Galectin-1 and 3 are the most commonly studied markers because they are important in different pathophysiological processes like cell growth, differentiation, adhesion, apoptosis, immune evasion, and neoplastic transformation.^7^

TROP-2 is a trophoblast surface antigen. It is a transmembrane glycoprotein of 35 kilodaltons (kDa) weight coded by a tumor-associated calcium signal transducer 2 (Tacstd2) gene first detected in trophoblastic cells, choriocarcinoma, and different normal tissues.^8^ It is regarded as a cell surface receptor contributing to many vital cellular events, such as increased intracellular calcium (Ca^2+^) levels.^8^

TROP-2 is regarded as an oncogene because it is found to be upregulated in many human cancers, like ovarian, pancreatic, prostatic, etc. TROP2 plays an important role in tumor cell proliferation, apoptosis, and invasion, thereby impacting the prognosis and treatment of cancer patients.^9^ The differentiation between benign and malignant thyroid neoplasms and between the most common subtypes of thyroid malignancy, namely the papillary and follicular thyroid carcinoma, remains a diagnostic challenge in many cases, especially those with overlapping morphological features. Several immunohistochemical markers, like CK19, HBME-1, and galectin-3, were extensively studied with inconsistent results.^10,11^

Therefore, the present study aimed to evaluate the IHC expression of galectin-1 and TROP-2 in differentiating benign versus malignant lesions and between papillary and follicular thyroid carcinoma.

## 2. METHODOLOGY

This retrospective observational study included a total of 136 thyroid specimens of different thyroid lesions, including 50 cases of follicular nodular disease (FND), 23 of follicular adenoma (FA), 37 cases of papillary carcinoma and 26 cases of follicular carcinoma collected from the archive of teaching laboratories of Al-Yarmouk Teaching Hospital for the period from October 2022 to October 2024 with prior ethical approval (no. 13/106 in 7/9/2022) and informed consent from the patients were obtained.

All the relevant clinical data (age, sex, tumor size, and lymph node [LN] involvement) were taken from the patient’s electronic pathological reports. Three expert pathologists reviewed the H&E slides to confirm the diagnosis.

### 2.1 Eligibility criteria

**2.1.1 Inclusion criteria**

1. Thyroid specimens with histologically confirmed differentiated thyroid lesions:
  - Follicular nodular disease (FND)
  - Follicular adenoma (FA)
  - Papillary thyroid carcinoma (PTC)
  - Follicular thyroid carcinoma (FTC)
2. Available clinical and pathological data, including patient age, sex, tumor size, and lymph node status.

**2.1.2 Exclusion criteria**

1. Poorly Differentiated Thyroid Carcinoma (PDTC)
  - This diagnosis was excluded due to very few available cases in the explored laboratory archives and because of the presence of well-developed criteria for diagnosing the poorly differentiated carcinoma (The Turin criteria). Moreover, the two markers studied in our study were mainly designed for the differentiation of well-differentiated follicular-derived thyroid lesions.
2. Medullary Thyroid Carcinoma (MTC)
  - This diagnosis was excluded from our study because these tumors originate from the parafollicular or C-cells and express specifically calcitonin and neuroendocrine markers and show amyloid deposits by the Congo Red stain.
3. Specimens with incomplete clinical or pathological data (e.g., missing age, tumor size, or lymph node status)
  - Regarding sample size, we recommend using a larger sample size to achieve a more robust statistical analysis. To minimize subjectivity in the interpretation of the results, all slides were independently reviewed and assessed by three pathologists. The selected tissue blocks were preserved in a designated storage room under controlled temperature and protected from light. In this study, antigen retrieval methods were applied to enhance antigen exposure. No discrepancies were observed between the initial and final diagnosis.

Appropriate and representative paraffin blocks from each case were chosen, and 5 μm-thick sections were cut on positively charged glass slides (PathnSitu Biotechnologies).

The immunohistochemical method used was similar to that described in our previous papers, for example, Gaidan et al., Hasan et al., and Al-Shami et al.^12–14^ some modifications were made, as summarized below.

The slides were de-paraffinized, and antigen retrieval was done by heat using a water bath in PBS for 45 minutes at 95°C. Then, slides were treated with 3% H_2_O_2_ for 15 minutes to block endogenous peroxidase and then incubated at room temperature with bovine serum albumin (BSA) to minimize non-specific staining. Overnight incubation with the following primary antibodies was done for each case:

Galectin-1 mouse monoclonal antibody (clone 6C8.4-1), Thermo Fisher Scientific™, 1:20 dilution, and TROP-2 (EGP-1) mouse monoclonal antibody (clone MR54), Thermo Fisher Scientific, 1:20 dilution. Appropriate positive control normal colon for galectin-1 and placental tissue for TROP-2, and a negative technical control by skipping primary antibody incubation were used with each run. A secondary antibody detection kit was used, and slides were mounted, examined, and assessed in a semi-quantitative way.

### 2.2 Immunohistochemical staining

Evaluation of TROP-2:

Continuous and strong membranous staining of more than 5% of cells was considered positive, and nuclear or cytoplasmic staining, if present, was neglected, similar to the interpretation criteria used by Simms et al.^15^

It was regarded as negative (no or <5% of tumor cells, 1+ (5%–25%), 2+ (26%–50%), 3+ (51%–75%), and 4+ (>75%) following the scoring criteria by Murtezaoglu and Gucer.^16^

**Figure 1:**
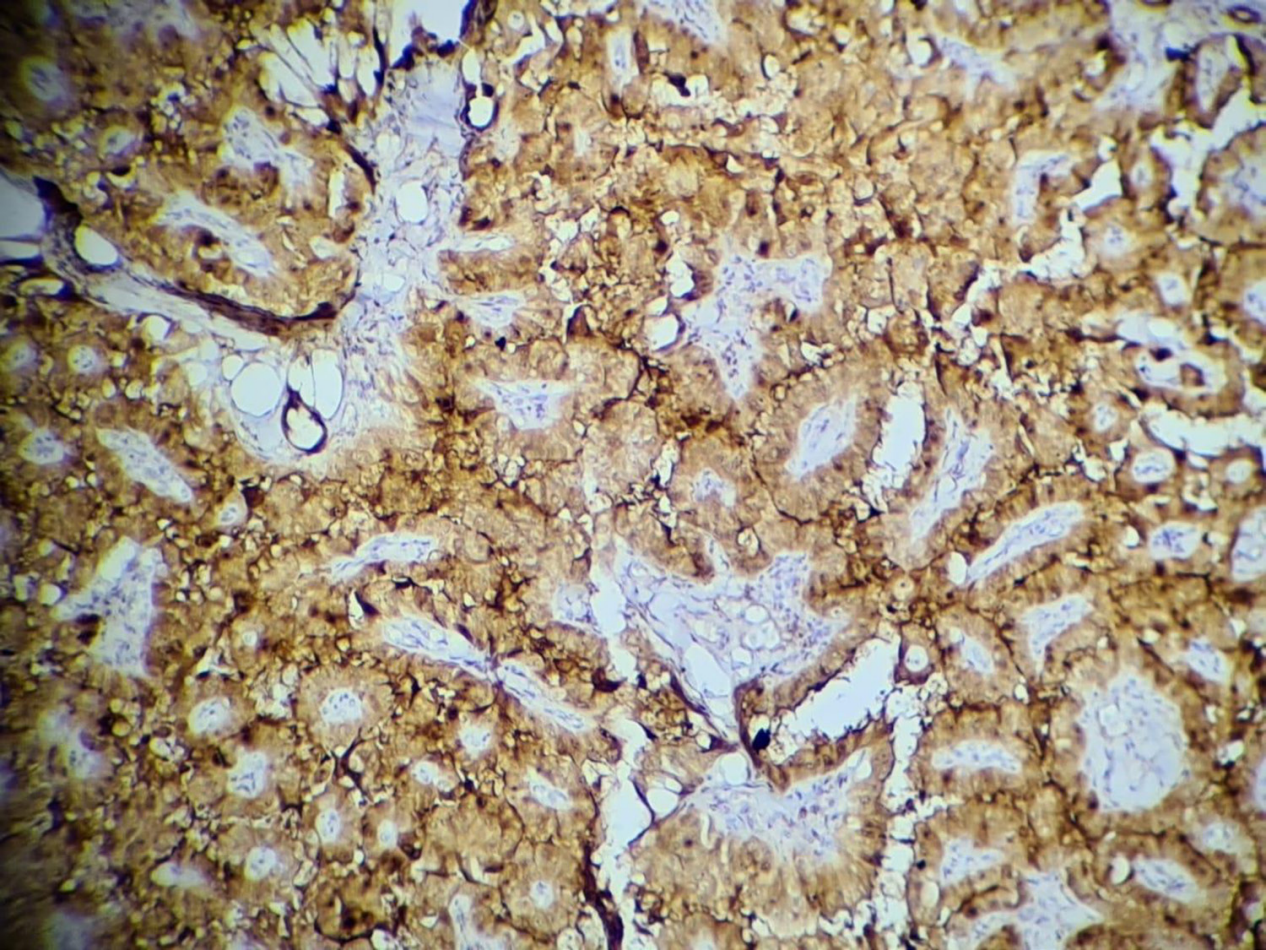
Papillary carcinoma of the thyroid showing strong cytoplasmic staining with galectin 1 (100X; immunohistochemistry).

**Figure 2:**
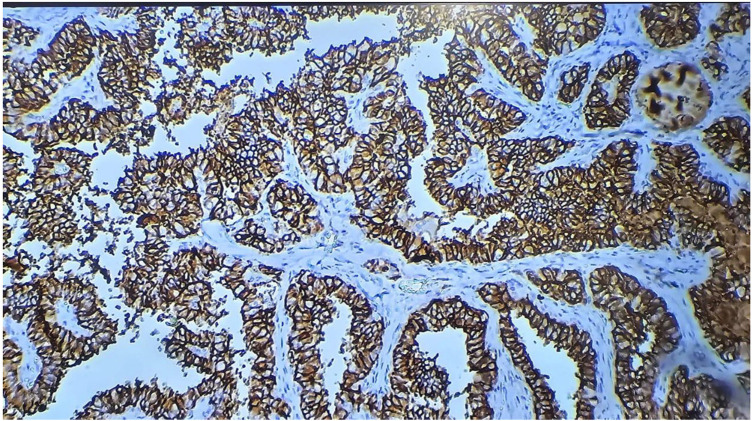
Papillary carcinoma of the thyroid showing strong membranous staining with TROP-2 (100X; immunohistochemistry).

**Figure 3:**
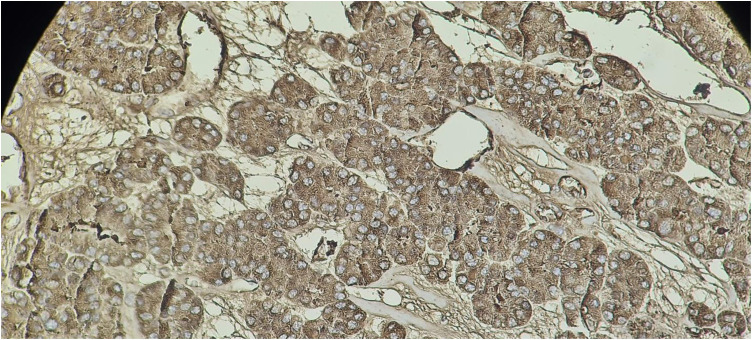
Follicular carcinoma of the thyroid showing strong cytoplasmic staining with galectin 1 (100X; immunohistochemistry).

**Figure 4:**
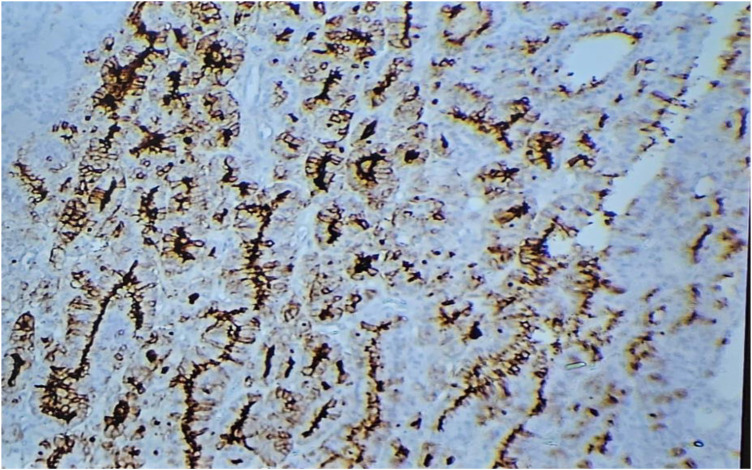
Follicular carcinoma of the thyroid showing strong membranous staining with TROP-2 (100X; immunohistochemistry).

Evaluation of galectin-1:

Staining for galectin-1 was observed in the cytoplasm of thyroid epithelial cells and was scored as: 0–3: 0% (no), 1 = 1%–33% (low), 2 = 34%–66% (moderate), and 3 = 67%–100% (high).^17^ See Figures 1 to 4.

### 2.3 Statistical analysis

The collected data were expressed as numbers, and percentages were compared.

Then they were coded, entered, presented, and analyzed by computer using the available database software program, the statistical package of IBM SPSS-29 (IBM Statistical Packages; Package for the Social Sciences, version 29, Chicago, IL). Data were presented in simple measures of frequency and percentages.

The significance of differences of different percentages (qualitative data) was tested using the Pearson chi-square test (*χ*^2^-test) with application of Yates’ correction or Fisher’s exact test whenever applicable. Statistical significance was considered whenever the P value was equal to or less than 0.05.

## 3. RESULTS

For all 136 cases, the results revealed the following.

### 3.1 Clinicopathological parameters

The age range for benign cases (FND and FA) was between 23 and 62 years, with a mean of 43 years.

In Papillary carcinoma, 48.6% (18/37) were <45 years, and 51.4% (19/37) of cases were ≥45 years. Regarding sex, 21.6% (8/37) of cases were males, while 78.4% (29/37) of cases were females. The tumor size: 45.9% (17/37) of cases were between 2 and 4 cm, 32.5% (12/37) cases were >4 cm, and only 21.6% (8/37) cases measured <2 cm.

Lymph node metastases: the majority of the cases, 83.8% (31/37), were negative, and only 16.2% (6/37) of cases were detected to be positive. Results are summarized in Table 1.

Regarding follicular carcinoma, 57.7% (15/26) of cases were ≥45 years, while 42.3% (11/26) of cases were <45 years. As for the sex, in most cases, 69.2% (18/26) were females, and only 30.8% (8/26) were males. Tumor size: 46.2% (12/26) were more than 4 cm, 30.8% (8/26) of cases were 2 to 4 cm, and 23% (6/26) of cases were <2 cm. Lymph node Metastases were not detected in FC cases.

### 3.2 Results of immunohistochemistry

Regarding benign cases:

Galectin-1 IHC staining in benign lesions was detected in 8% (4/50) of FND cases, while 17.4% (4/23) of cases of FA were stained with galectin-1.

TROP-2 IHC staining in benign cases showed that 16% (8/50) of FND expressed TROP-2, while 21.7% (5/23) of cases of FA were positive for TROP-2. The results are shown in Table 3.

Results of IHC staining in papillary carcinoma:

Galectin-1 IHC staining in papillary carcinoma: 62.2% (23/37) of cases were positively stained, 37.8% (14/37) of cases were negative.

There was a significant difference in IHC of galectin-1 in papillary carcinoma between different tumor sizes, where all cases that were ≥4 cm (100%) showed expression for galectin-1 (P value, 0.002). There was no significant difference between age, sex, and lymph node metastases in papillary carcinoma and the degree of staining.

However, all cases of papillary carcinoma associated with lymph node metastasis were positively stained with galectin-1, while 54.8% (17/31) of negative lymph node metastasis cases were positive for the marker, and 45.2% (14/31) of cases with no lymph node metastasis were negative for galectin-1.

Results are summarized in Table 1.

TROP-2 IHC staining in papillary carcinoma: TROP-2 was stained positively in 89.2% (33/37) of cases. There was a significant difference between females, 96.6% (28/29), and males, 62.5% (5/8), *P* = 0.006. There was no significant difference in staining with age, tumor size, or lymph node metastasis. See Table 2.

Results of IHC staining in follicular carcinoma:

Galectin-1 IHC staining in follicular carcinoma: galectin-1 was stained positively in 73.1% (19/26) of cases, while 7 cases were negative 26.9% (7/26).

There was no significant difference in the IHC of galectin-1 in follicular cancer with age, sex, or tumor size. Results are summarized in Table 1.

IHC of TROP-2 in follicular carcinoma: 73.1% (19/26) of cases were stained positively for TROP-2. There was a significant difference in IHC staining of TROP-2 in follicular carcinoma in different sizes: of tumors ≥4 cm, 91.6% (11/12) were strongly stained with this marker (*P* = 0.031). Results are summarized in Table 2.

IHC of galectin-1 and TROP-2 in benign lesions: 8% (4/50) of FND cases were positively stained for galectin-1, and 16% (8/50) cases were positively stained for TROP-2.

17.4% (4/23) of follicular adenomas were positive for galectin-1, and 21.7% (5/23) were positively stained for TROP-2 (results are summarized in Table 3).

Galectin-1 IHC staining and its difference between benign (FND and FA) versus malignant lesions (papillary and follicular carcinoma):

62% (39/63) of malignant lesions (papillary and follicular carcinoma) were stained positively with galectin-1 compared to 11% (8/73) of benign lesions (FND and FA), which were stained positively for this marker; the P-value was significantly different (*P* = 0.0001) shown in Table 3.

TROP-2 IHC staining and its difference between benign (FND &FA) versus malignant lesions (papillary and follicular carcinoma):

82.5% (52/63) of malignant cases (papillary and follicular carcinoma) were positively stained compared to only 17.8% (13/73) of benign cases (FND and FA), which were stained positively for this marker with a highly significant difference (*P* = 0.0001). There was a significant difference between the two markers in malignant cases, where 82.5% (52/63) were positively stained for TROP-2 in comparison to only 61.9% (39/63) of malignant cases which were positively stained for galectin-1 (*P* = 0.0097). Results are summarized in Table 3.

The sensitivity and specificity of both markers were compared in Table 4:

Galectin-1: sensitivity was 61.9%, while its specificity was 89.0%

TROP-2: Its sensitivity was 82.5%, while its specificity was 82.2 %.

## 4. DISCUSSION

The gold standards that pathologists usually use to differentiate between benign versus malignant thyroid lesions are histopathological examination using hematoxylin and eosin-stained slides and the fine needle aspiration (FNA) technique.

However, there is an increasing need to use more accurate methods like IHC markers to improve diagnostic abilities. No single IHC marker is sensitive or specific enough to be used for this task. This study investigates the importance of the two immunohistochemical markers (galectin-1 and TROP-2) in differentiating benign and malignant thyroid lesions and between follicular and thyroid carcinoma.

This study shows a significant difference between the tumor size of papillary carcinoma and galectin-1 staining, where all cases ≥ 4 cm were positively stained for galectin-1 with P-value = 0.002, while TROP-2 shows a significant difference in staining of follicular carcinoma with tumor size ≥4 cm (*P*-value, 0.03).

Our results are in agreement with those of Guan et al., who demonstrate that positive TROP-2 expression correlates with TNM staging and nodal classification and is subsequently associated with poor prognosis of thyroid malignancy, as it is associated with advanced tumor stage.^6,7,14,15,18,19^

The current study results show that all 16.2% (6/37) cases of papillary carcinoma with lymph node metastases are positively stained for galectin-1 (100%). However, this was not statistically significant, which may be related to the small number of cases. This result agrees with Salajegheh et al. and Kim et al., who found galectin-1 to be overexpressed in papillary carcinoma cases associated with lymph node metastases in comparison with cases lacking lymph node metastasis, suggesting its role in the progression of papillary carcinoma from the primary site to nearby lymph nodes.^19,20^

The tumor may express galectin-1 to increase its ability to bind extracellular matrix in blood vessels and distant tissues, which will increase the metastatic potential of the tumor.^15,16,19,20^

Galectin-1 is a potential target for treatment to prevent tumor metastases, as it has a role in the transmission of tumors from primary locations to widespread metastases.

Regarding TROP-2, 83.3% of 5 of 6 cases, which were associated with lymph metastases, were stained positively for TROP-2, which was not statistically significant (also may be related to a small number of cases, but this result is in agreement with Abdou et al. and Guan et al., who found a significant association with of TROP-2 positivity with lymph node metastases. A possible explanation for this finding is that TROP-2 was found to potentiate the invasion and migration of thyroid tumor cells through activation of protein 1 (AP-1), leading to matrix metalloproteinase upregulation involved in the destruction of type IV collagen and assisting in tumor spread via the extracellular matrix.^8,21^

It was reported that high expression of TROP-2 can be used as a potential marker for lymph node metastases in papillary carcinoma, unlike follicular carcinoma, which metastasizes hematogenously.^22^

Benign versus malignant lesions:

There was a highly significant difference in IHC expression of galectin-1 and TROP-2 in benign (FND and FA) versus malignant lesions (*P*-value = 0.0001).

There was a highly significant difference in galectin-1 expression between 11% (8/73) of benign versus 62% (39/63) of malignant cases (*P*-value = 0.0001).

Our results support that galectin-1 is an important diagnostic marker for thyroid carcinoma, given its involvement in cell migration, proliferation, tumor growth, and invasion. This suggests its critical role in thyroid cancer progression and its potential target in cancer therapy.^17^

TROP-2 shows a highly significant difference in IHC expression between benign and malignant cases: 18% (13/73) versus 82% (52/63) with P-value = 0.0097; this was in agreement with previous studies (Abdou et al., Addati et al., and Abu-Seadah et al.).^21,23,24^

The results of this study report the important role of TROP-2 as a potential marker in the differential diagnosis of benign and malignant lesions that arise from follicular epithelial cells.^16^

Whereas TROP-2 IHC expression in papillary carcinoma was 89.2% (33/37), which approximates the results of other studies by Abdou et al. (71.4%), Bychkov et al. (81.5%), and Liu et al. (81.6%).^21,25,26^

Our results support previous studies considering TROP-2 a reliable marker for diagnosing papillary carcinoma and its differentiation from non-neoplastic lesions with papillary architecture.^25,26^

This study shows that TROP-2 in follicular carcinoma is 73.1% (19/26), which approximates the results of Abu-Seadah et al. (71.4%) and Zargari and Mokhtari (83.3%), and supports its usefulness in distinguishing follicular carcinoma from Papillary carcinoma and its variants, especially those with solid components.^24,27^

Sensitivity and specificity:

This study shows galectin-1 exhibits excellent specificity (89.0 %) to differentiate benign from malignant thyroid lesions, which approximates the results of Fanfone et al., who showed 100% specificity and 75%sensitivity, Arcolia et al. (sensitivity of 80% and specificity 97%), and a second study by Fanfone et al., who found 80% sensitivity and 100% specificity and on fine needle aspiration samples.^1,17,28^

TROP-2: Its sensitivity is 82.5% while its specificity is 82.2 %; this is in agreement with Arcolia et al. (sensitivity 83% and specificity 80%), Abdou et al. (sensitivity 71% and 81% specificity for the total diagnosis of Papillary Thyroid Carcinoma, Bychkov et al. (sensitivity 75% and specificity 98.4%), Liu et al. (TROP-2 sensitivity is 94% for classic papillary thyroid carcinoma and 81% for confirmation of follicular variant of papillary thyroid carcinoma.^17,21,25,26^

These data show that membranous staining of TROP-2 is specific for both classic and follicular variants of PTC, while Zargari and Mokhtari (sensitivity of 93% and specificity of 74%) between benign and malignant lesions, and 83.3% of follicular carcinomas, 95.2% of follicular variant of papillary carcinomas, who reveals strong membranous staining for TROP-2.^27^

Others show that TROP-2 appears to have a strong role in differentiating non-neoplastic lesions such as Graves’ disease, hyperplastic nodules in FND, and hyperfunctioning adenoma from classic PTC but not the follicular variant of PTC.^22^

According to these results, a combination of immunohistochemical markers like galectin 1 and TROP-2 can be recommended as initial panels to help accurately diagnose thyroid follicular lesions with challenging morphological features.

Sensitivity and specificity of both markers:

Galectin-1 immunohistochemical expression shows sensitivity (61.9%), which is lower than the sensitivity of TROP-2 (82.5%); other studies done by Abdou et al., Abu-Seadah et al., and Eid and Abo Safia show that TROP-2 is highly sensitive (71%, 98.1%, 87.78%) in the detection of papillary thyroid carcinoma, respectively.^21,24,29^

The specificity of both markers is nearly similar, with a slightly higher value for galectin-1 (89%) in comparison to TROP-2 (82.2%), which is in agreement with Abdou et al. and Abu-Seadah et al. (81%, 97.5%).^21,24^

The results of this study regarding the sensitivity and specificity of galectin-1 (61.9% sensitivity and specificity of 89%) are within the same range as one of the most commonly used markers in routine work (galectin-3) and agree with the results of Vella et al. (sensitivity of 95% and specificity of 92.5%), Bhandari et al. (sensitivity of 97.92% and specificity of 50.^30,31^

This variation in the results could be caused by using galectin-1 instead of galectin-3 in their studies, but we use this type of galectin to study its efficiency in differentiating benign from malignant lesions; also, we can use galectin-1 in routine work if galectin-3 is unavailable. It could also be caused by immunostaining techniques, staining scoring methods, and sample size differences.

### 4.1 Study limitations

A multicentric study would perform better as its results would be liable for generalization. The lack of long-term prognostic correlations is another limitation; the current study has focused on IHC expression without evaluating its impact on the long-term prognosis of the patients.

### 4.2 Study strengths

The study recruited a good and diverse sample size that covered both benign and malignant lesions, enhancing the validity and generalizability of its findings. Additionally, the robust statistical analysis showed the importance of both markers in improving diagnostic accuracy in practice.

## 5. CONCLUSION

Galectin-1 and TROP-2 were highly expressed in malignant cases compared to benign lesions. TROP-2 is more sensitive in detecting malignant lesions, while galectin-1 is more specific. Both markers are essential for differentiation between benign and malignant lesions.

### 5.1 Recommendations

Future multicentric studies should examine the prognostic value of these markers in predicting disease progression and therapeutic responses. Integrating IHC markers with molecular profiling may refine thyroid cancer classifications and management strategies.

## ETHICAL APPROVAL

The protocol was approved 38 by the Mustansiriyah Ethics Committee of Baghdad /Iraq (NO.13/106 in 7/9/2022). This study was approved by the Institutional Scientific Committee of the College of Medicine, Mustansiriyah University. Baghdad /Iraq (NO.13/106 in 7/9/2022).

## AUTHOR’S CONTRIBUTION

All authors contributed equally to the manuscript’s conception, literature search, writing, editing, and revising. All authors read and agreed on the final version.

## ACKNOWLEDGEMENTS

We would like to thank Mustansiriyah University for its continuous support.

## INFORMED CONSENT

All patients gave their signed consent before participating in the study.

## CONFLICT OF INTEREST

The authors declare that there was no conflict of interest.

## Figures and Tables

**Table 1.** Immunohistochemical expression of galectin-1 in PC and FC and its association with clinicopathological parameters.

	Galectin-1 in papillary carcinoma				Galectin-1 in follicular carcinoma			
	No. of cases	Positive (*n* = 23)	Negative (*n* = 14)	*P* value	No. of cases	Positive (*n* = 19)	Negative (*n* = 7)	*P* value
Age								
<45 years	18	10 (55.6%)	8 (44.4)	0.420	11	(81.8%)	2 (18.2%)	0.390
≥ 45 years	19	13 (68.4%)	6 (31.6%)		15	10 (66.7%)	5 (33.3%)	
Sex								
Male	8	5 (62.5%)	3 (37.5%)	0.982	8	5 (62.5%)	3 (37.5%)	0.418
Female	29	18 (62.1%)	11 (37.9%)		18	14 (77.8%)	4 (22.2%)	
Tumor size								0.671
≤2 cm	8	5 (62.5%)	3 (37.5%)	0.002*	6	5 (83.3%)	1 (16.7%)	
2–4 cm	17	6 (35.3%)	11 (63.6%)		8	5 (62.5%)	3 (37.5%)	
>4 cm	12	12 (100%)	0 (0%)		12	9 (75.0%)	3 (25.0%)	
Lymph node metastases								-
Present	6	6 (100%)	0 (0%)	0.065	0	0 (0%)	0 (0%)	
Absent	31	17(54.8%)	14 (45.2%)		26	21 (80.8%)	5 (19.2%)	

*Significant difference between percentages using the Pearson chi-square test (*χ*^2^-test) at the 0.05 level.

**Table 2.** Immunohistochemical expression of TROP-2 in PC and FC and its association with clinicopathological parameters.

	TROP-2 in papillary carcinoma				TROP-2 in follicular carcinoma			
	No. of cases	Positive (*n* = 33)	Negative (*n* = 4)	*P* value	No. of cases	Positive (*n* = 19)	Negative (*n* = 7)	*P* value
Age								
<45 years	18	16 (48.5%)	2 (50%)	0.954	11	8 (72.7%)	3 (27.3%)	0.973
≥45 years	19	17 (51.5%)	2 (50%)		15	11 (73.3%)	4 (26.7%)	
Sex								
Male	8	5 (62.5%)	3 (37.5%)	0.006*	8	6 (75.0%)	2 (25.0%)	0.883
Female	29	28 (96.6%)	1 (3.4%)		18	13 (72.2%)	5 (27.8%)	
Tumor size								
<2 cm	8	6 (75.0%)	2 (25.0%)		6	2 (33.3%)	4 (66.6%)	
2–4 cm	17	16 (94.1%)	1 (5.9%)	0.337	8	6 (75.0%)	2 (25.0%)	0.031*
>4 cm	12	11 (91.6%)	1 (8.3%)		12	11 (91.6%)	1 (8.3%)	
Lymph node metastases								-
Present	6	5 (83.3%)	1 (16.6%)	0.614	0	0 (0%)	0 (0%)	
Absent	31	28 (90.3%)	3 (9.7%)		26	19 (73.1%)	7(26.9%)	

*Significant difference between percentages using the Pearson chi-square test (*χ*^2^-test) at the 0.05 level.

**Table 3.** Comparison between immunohistochemical expression results in benign and malignant tissues and their difference between the two markers (Galectin-1 versus TROP-2).

Diagnosis	Galectin-1		TROP-2		*P*-value between two markers
	Positive	Negative	Positive	Negative	
Follicular nodular disease (FND) and follicular adenoma (FA; *n* = 73)	8 (11%)	65	13 (18%)	60	0.239
Papillary carcinoma (PC) and follicular carcinoma (FC; *n* = 63)	39 (62%)	24	52 (82%)	11	0.0097*
*P* value between (FND + FA) Vs. (PC + FC)	0.0001*		0.0001*		

*Significant difference between percentages using the Pearson chi-square test (*χ*^2^-test) at the 0.05 level.

**Table 4.** Sensitivity and specificity of both markers.

IHC	Sensitivity	Specificity	PPV	NPV	Accuracy rate
Galectin-1	61.9%	89.0%	83.0%	73.0%	76.5%
TROP-2	82.5%	82.2%	80.0%	84.5%	82.4%

PPN, Positive predictive value; NPV, Negative predictive value.
